# Recombinant Expression and Characterization of α-Conotoxin LvIA in *Escherichia coli*

**DOI:** 10.3390/md14010011

**Published:** 2016-01-05

**Authors:** Xiaopeng Zhu, Jianpeng Bi, Jinpeng Yu, Xiaodan Li, Yaning Zhang, Dongting Zhangsun, Sulan Luo

**Affiliations:** 1 Key Laboratory of Tropical Biological Resources, Ministry of Education, Key Laboratory for Marine Drugs of Haikou, Hainan University, Haikou 570228, China; zhuxiaopeng@hainu.edu.cn (X.Z.); jianpeng_bee@yahoo.com (J.B.); mtying0001@126.com (J.Y.); lixiaodan816@163.com (X.L.); yanin802388@yahoo.com (Y.Z.); zhangsundt@163.com (D.Z.); 2 College of Horticulture and Landscapes, Hainan University, Haikou 570228, China; 3 College of Marine Science, Hainan University, Haikou 570228, China

**Keywords:** α-conotoxin LvIA, recombinant expression, fusion protein, nAChRs, electrophysiology, pain assay

## Abstract

α-Conotoxin LvIA is derived from *Conus lividus*, native to Hainan, and is the most selective inhibitor of α3β2 nicotinic acetylcholine receptors (nAChRs) known to date. In this study, an efficient approach for the production of recombinant α-Conotoxin LvIA is described. Tandem repeats of a LvIA gene fragment were constructed and fused with a KSI gene and a His_6_ tag in a *Escherichia coli* (*E. coli*) expression vector pET-31b(+). The recombinant plasmids were transformed into *E. coli* and were found to express well. The KSI-(LvIA)_*n*_-His_6_ fusion protein was purified by metal affinity chromatography and then cleaved with CNBr to release recombinant LvIA (rLvIA). High yields of fusion protein ranging from 100 to 500 mg/L culture were obtained. The pharmacological profile of rLvIA was determined by two-electrode voltage-clamp electrophysiology in *Xenopus laevis* oocytes expressing rat nAChR subtypes. The rLvIA antagonized the α3β2 nAChR subtype selectively with a nano-molar IC_50_. The rLvIA was analgesic in a mouse hot-plate test model of pain. Overall, this study provides an effective method to synthesize α-conotoxin LvIA in an *E. coli* recombinant expression system, and this approach could be useful to obtain active conopeptides in large quantity and at low cost.

## 1. Introduction

*Conus* is a large genus that contains more than 500 different species of predatory marine cone snails. Cone snails are usually divided into three categories according to their prey, and include vermivorous (worm-hunting), molluscivorous (snail-hunting) and piscivorous (fish-hunting) species. *Conus* venoms are rich sources of bioactive small peptides that are known as conopeptides or conotoxins (CTxs). Each *Conus* species typically contains ~100 different peptides in the venom [1]. Conopeptides are also distinctive by virtue of their rich cysteine content, small size (10~40 aa) and high degree of post-translational modifications. Conopeptides fall into two main categories: disulfide-rich conopeptides (referred to a conotoxins) and peptides that lack multiple disulfides. In terms of the signal sequences and pattern disulfide linkages, conopeptides have been divided into several superfamilies, such as O-, M-, A-, S-, T-, P-, I- *etc.* The superfamily may be subdivided into several families with distinct pharmacological activities, for example the A-superfamily (α-, αA-, κA- and ρ-conopeptides), M-superfamily (μ-and ψ-conopeptides), O-superfamily (ω-, μO, δ-, and κ-conopeptides), T-superfamily (τ- and χ-conopeptides) and so on [2]. Individual conopeptides are selectively targeted to specific isoforms of receptors or ion channels.

α-Conotoxins (α-CTxs) are one of the most important conopeptide families and are known to target muscle and neuronal subtype nicotinic acetylcholine receptors (nAChRs). α-Conotoxins are the smallest conopeptides consisting of 12–19 amino acid residues [3]. They have a typical CC-Xm-C-Xn-C cysteine framework, where four cysteines can form three possible disulfide bridge isoforms, *i.e.*, globular (I-III,II-IV), beads (I-II,III-IV) or ribbon (I-IV,II-III). Native α-conotoxins usually exhibit the globular conformation. There is a further sub-classification of α-CTxs based on the number of residues in their two inter-cysteine loops (m/n: 3/5, 4/3, 4/4, 4/5, 4/6 and 4/7) [4]. Table 1 reflects the majority of α-conotoxin sequences and targets known to date. Due to their extraordinary pharmacological properties, conopeptides are increasingly used as probes in neuroscience, pharmacology research, and even in clinical trials [5]. From their natural sources, conopeptides can only be obtained in tiny quantities, limiting their availability for research and applications. To obtain larger amounts of conopeptides, two basic approaches are available, *i.e.*, chemical synthesis or recombinant production in heterologous expression systems [6,7,8]. The chemical methods are cost-consuming and associated with potential health risks, and, therefore, an alternative production method is needed to meet the high demand for conopeptides for investigations and clinical experiments.

Recombinant expression technology is a promising approach for the mass production of conopeptides. Our lab recently characterized a novel α-conotoxin LvIA (α-CTx LvIA, LvIA) from *Conus lividus* [9], which is the most selective inhibitor of α3β2 nAChRs to date. The precursor gene of LvIA has deposited in GenBank with accession number of HF566436. Here, we describe an efficient method to biosynthesize recombinant α-CTx LvIA (rLvIA) using an *E. coli* expression system.

**Table 1:** Sequence and receptor specificity of neuronal-specific α-conotoxins.

α m/n	Name	Species	Sequence	Target	Reference
α 3/5	GI	*C. geographus*	ECCNPACGRHYSC *	α1β1γδ	[10]
	MI	*C. magus*	GRCCHPACGKNYSC *	α1β1γδ	[11]
α 4/3	ImI	*C. imperialis*	GCCSDPRCAWRC *	α3β2, α7	[12]
	ImII	*C. imperialis*	ACCSDRRCRWRC *	α7	[12]
α 4/4	BuIA	*C. bullatus*	GCCSTPPCAVLYC *	α6/α3β2, α6/α3β4, α3β2, α3β4	[13]
α 4/6	TxIA	*C. textile*	GCCSROOCIANNPDLC *	α3β2, α7	[14]
	TxID	*C. textile*	GCCSHPVCSAMSPIC *	α3β4, α6/α3β4	[15]
α 4/7	LvIA	*C. lividus*	GCCSHPACNVDHPEIC *	α3β2, α3β4, α6β4	[9]
	GIC	*C. geographus*	GCCSHPACAGNNQHIC *	α3β2	[16]
	Vc1.1	*C. victoriae*	GCCSDPRCNYDHPEIC *	α9α10	[17]

* Denotes an amidated C-terminus; O, 4-*trans*-hydroxyproline; The cysteine residues that form the disulfide bridges are in bold.

## 2. Results

### 2.1. Construction of the LvIA Recombinant Vector

**Figure 1:**
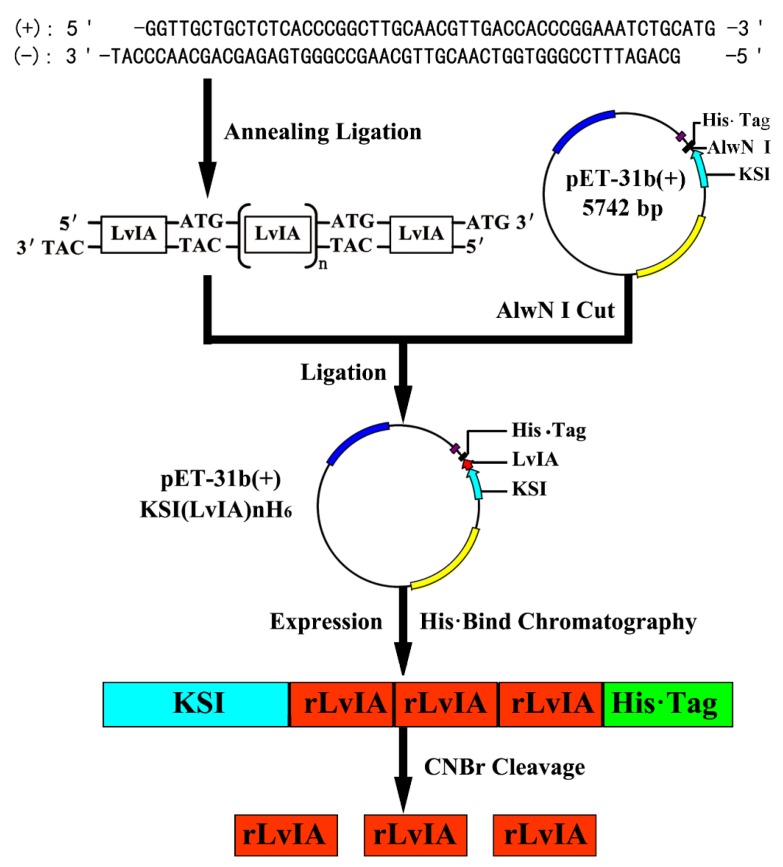
Strategy for construction, expression, purification and cleavage of fusion protein of recombinant α-conotoxin LvIA (rLvIA). Two primers encoding α-conotoxin LvIA mature peptide were annealed and unidirectionally self-ligated to form an array of tandem repeats. These multimers were then ligated into the *Alw*NI digested vector pET31b(+) to construct an array of recombinant KSI(rLvIA)_n_His_6_ vectors, where *n* = 1–11. These gene tandem repeats fused with KSI and His·Tag were expressed in *E. coli* after the addition of IPTG. The fusion proteins were purified by Ni^2+^ chromatography and cleaved by CNBr into individual soluble rLvIA, insoluble KSI, and the 6× His·tag tail.

**Figure 2:**
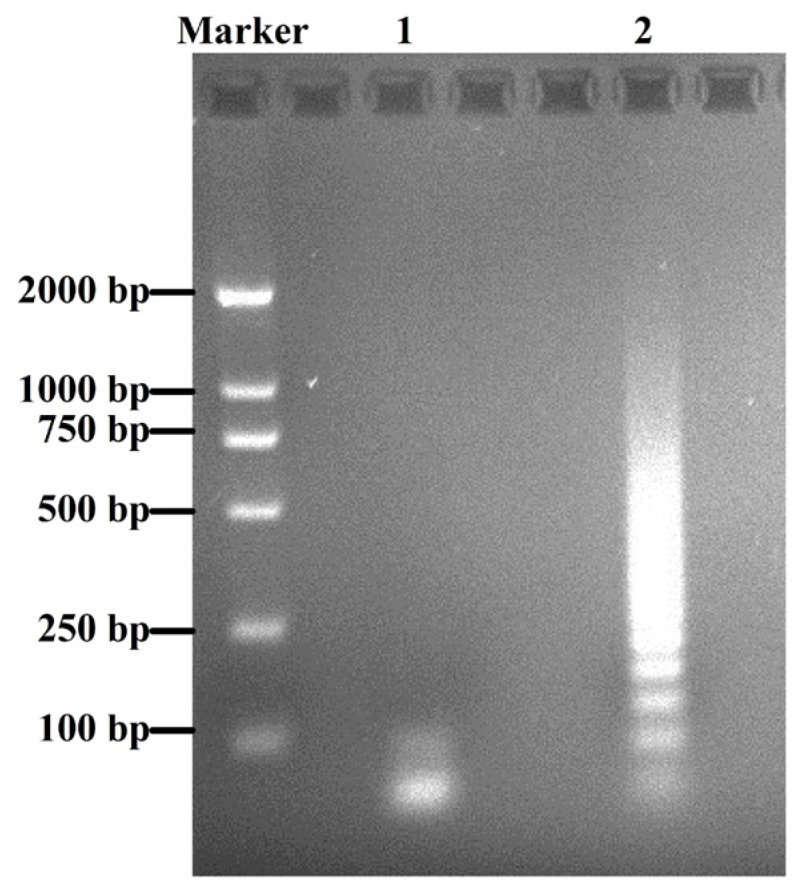
Agarose gel electrophoresis analysis of the LvIA gene tandem repeats. Two phosphorylated oligos were annealed and ligated. These oligos could be ligated to form array of tandem repeats. These multimers were analyzed by 3% agarose gel electrophoresis. Marker, DL 2000 DNA marker. Lane 1, annealed single gene with 51 bp. Lane 2, tandem repeats of rLvIA gene with different size, ranged from 51 to 1000 bp.

The pET-31b(+) expression system designed for the production of small peptides in *E. coli* was used to express α-conotoxin LvIA. The sense and anti-sense oligonucleotide strands encoding α-CTx LvIA were designed and synthesized respectively using the preferred codon usage of *E. coli* according to the mature peptide gene sequence [9] (GenBank: HF566436), which facilitated LvIA expression in *E. coli* (Figure 1). Complementary oligonucleotides encoding α-CTx LvIA were annealed, and self-ligated to form an array of tandem repeated units. The sizes of these repeats ranged from 100 to 1000 bp, which were examined using 3% agarose gel electrophoresis (Figure 2). The expression vector was digested with endonuclease *Alw*NI, which recognizes CAGNNN↓CTG. Each different size tandem repeat was ligated with the *Alw*NI digestion vector, and was inserted downstream of a highly expressed KSI gene of 125 amino acids and upstream of a His_6_·Tag sequence to create an array of pET-31KSI(LvIA)_*n*_His·Tag constructs (*n* = 1–11). The numbers of multimers were determined by sequencing in Sangon Co. (Shanghai, China).

### 2.2. Expression and Purification of Recombinant Protein

**Figure 3:**
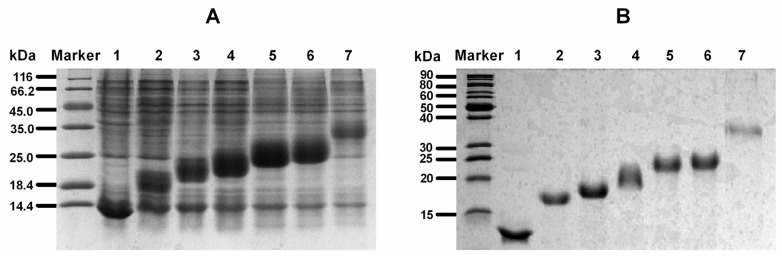
SDS-PAGE analysis of recombinant KSI(rLvIA)_n_His_6_ fusion proteins in *E. coli.* (**A**) Cell total proteins expressed by IPTG induction. Lane 1–7 were total protein fractions containing KSI(rLvIA)_*n*_His_6_ fusion constructs, where lane 1–6, *n* = 0–5. lane 7, *n* = 11. (**B**) Recombinant fusion proteins from panel A cell total proteins purified by Ni^2+^ Chelation Chromatography containing different rLvIA tandem repeats.

All the pET-31(LvIA)_*n*_His·Tag constructs were transformed into protease deficient host strain BLR(DE3)plysS for protein expression, which is a *recA*^−^ derivative of BL21 and is potentially superior for stabilizing tandem repeats. The fusion proteins with different LvIA repeats were expressed (Figure 3A) and purified from inclusion bodies expressed in *E. coli* by Ni affinity chromatography (Figure 3B). Since the KSI fusion partner is insoluble, the target fusion proteins were found in the insoluble fraction and processed into inclusion bodies. The fusion proteins could be dissolved completely in 1× Binding Buffer solution which contained 8 M urea or 6 M guanidine-HCl. The expression level of fusion proteins reached a maximum by introduction of 1 mM of IPTG at ~7 h. The inclusion body protein pellets were dissolved in 8 M urea solution and purified by Ni metal chelation chromatography. All the target fusion proteins were eluted with Elution Buffer containing 250 mM imidazole. The purified recombinant proteins were analyzed by 12% SDS-PAGE (Figure 3B). The molecular weight of fusion proteins ranged from 14 kDa to 40 kDa. Fusion proteins were dialyzed and concentrated to yield products with high concentration of as high as 500 mg/L.

### 2.3. CNBr Cleavage and Purification of Recombinant Protein

**Figure 4:**
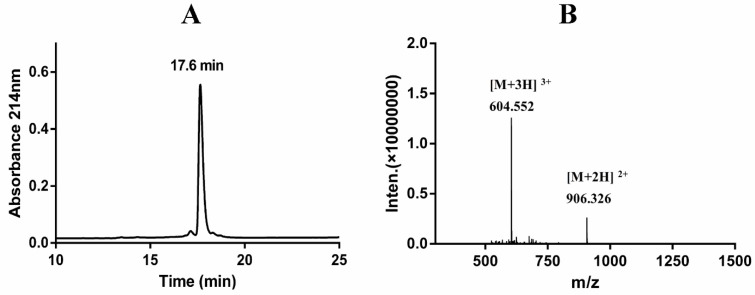
RP-HPLC chromatogram (**A**) and ESI-MS data (**B**) of rLvIA. (**A**) The peptide rLvIA was analyzed by RP-HPLC on a Vydac C18 column (5 μm, 4.6 mm × 250 mm), using a linear gradient of 0%–40% Buffer B over 20 min, where A = 0.075% TFA and B = 0.75% TFA, 90% acetonitrile, and the remainder water. Absorbance was monitored at 214 nm. (**B**) ESI-MS analysis of rLvIA with observed molecular weight of 1810.656 Da, which was consistent with the calculated theoretical molecular weight of 1810.635 Da.

The lyophilized fusion protein was dissolved in 70% of formic acid to a concentration of 10 mg/mL. CNBr was added to the protein solution at 100-fold molar excess of the concentration of Met residues in the fusion protein. CNBr specifically cleaved the fusion protein at the Met junction to release insoluble KSI, the His_6_ tag, and rLvIA simultaneously. The rLvIA was easily extracted from hydrophobic KSI using 40% CH_3_CN/60% H_2_O/0.1% TFA and purified by RP-HPLC. The retention time of rLvIA in the HPLC profile was 17.6 min (Figure 4A). The observed molecular weight of rLvIA by ESI-MS analysis was 1810.656 Da which is consistent with its theoretical molecular weight of 1810.635 Da (Figure 4B). The monoisotopic mass of rLvIA was 4 Da less than the linear peptide mass (1814.66 Da), which confirmed the formation of two disulfide bonds by air oxidation. For α-conotoxins, the globular conformation with a 1–3, 2–4 Cys connectivity is the native form and usually the bioactive conformation [18].

### 2.4. Bioassay of rLvIA in Pain Model

**Figure 5:**
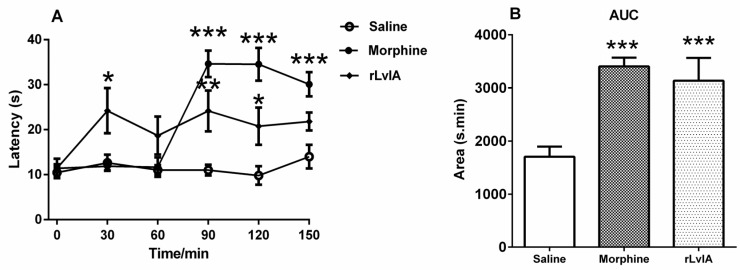
Analgesic effect of rLvIA on a mouse hot-plate test model. (**A**) Effect of rLvIA (10 nmol), 0.9% saline, and morphine (250 nmol) on 55 °C hot plate test at 30, 60, 90, 120, 150 min after injection as described in *Materials and Methods*. Latency time (second, s) was used as a measure of thermal hyperalgesia of conopeptide rLvIA (1 nmol/μL). Mice were divided into three groups, each group containing eight mice (*n* = 8). rLvIA significantly increased the latency time to the thermal stimulus at 30, 90, 120 min after treatment (*p* < 0.05). The maximum effect of rLvIA was observed at 90 min, which showed a latency of 24.17 s. (**B**) Corresponding dose response calculated as area under the curve (AUC) for data from each drug in (**A**) for time points between 0 and 150 min. Asterisks represent significant difference from the saline control group (*p < 0.05, **p < 0.01, ***p < 0.001).

The bioactivity of rLvIA in a hot-plate pain model was assessed with Kunming mice. The antinociceptive effects of rLvIA and morphine against thermal stimuli in the hot-plate test are given in Figure 5A. At 10 nmol dose, rLvIA significantly increased the reaction time (latency) to the thermal stimulus at 30, 90, 120 min after treatment (*p* < 0.05). The maximum effect of rLvIA was observed at the 90 min time point, which showed a latency time of 24.17 s. Morphine showed highest latency at 90, 120, 150 min after treatment (*p* < 0.001). The area under the curve (AUC) was calculated for each treatment: rLvIA displayed a significant increase in AUC (*p* < 0.01), which did not significantly differ from the effect of 250 nmol of morphine (*p* > 0.05) (Figure 5B).

### 2.5. Effect of rLvIA on ACh-Evoked nAChR-Mediated Currents

**Figure 6:**
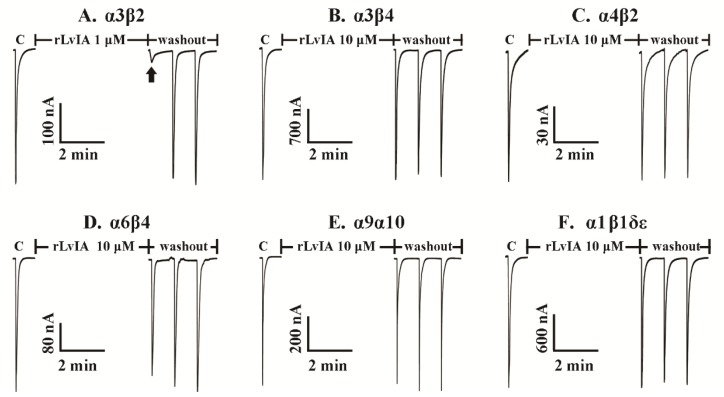
rLvIA selectively blocks α3β2 subtype among the tested nAChRs. nAChR subtypes were expressed as described in Materials and Methods. “C” indicates control responses to ACh. Oocytes were then exposed to 1 μM or 10 μM rLvIA for 5 min, followed by application of a 1 s pulse of ACh. The rLvIA almost blocked α3β2 at 1 μM concentration completely (arrow) (**A**), and the current resumed quickly within one minute of toxin wash out, For other nAChR subtypes even 10 μM rLvIA had little or no blockage (**B**–**F**).

**Figure 7:**
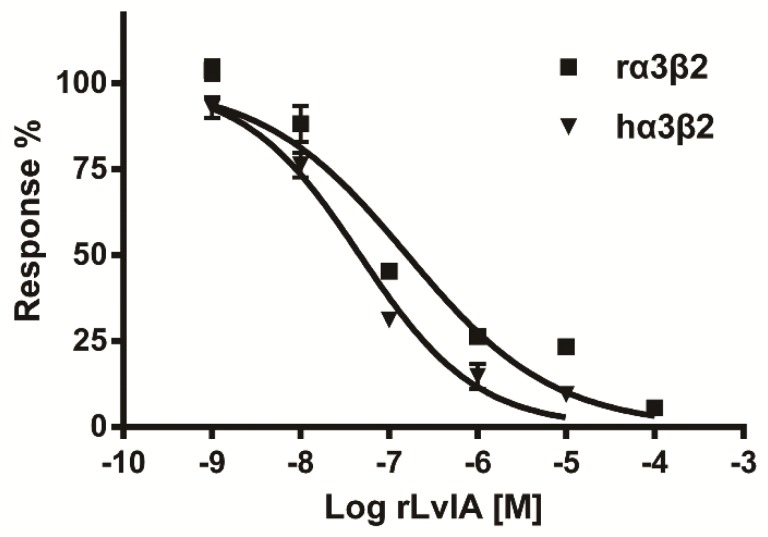
rLvIA concentration-response data for human and rat α3β2 nAChR subtypes. Oocytes expressing human and rat α3β2 nAChR subtypes were voltage clamped and subjected to ACh pulses as described in the *Experimental Procedures*. Values given are mean ± SEM from 5 to 8 separate oocytes. The IC_50_ values for rLvIA on hα3β2 and rα3β2 were 46.8 nM and 160.8 nM, respectively.

The rLvIA was tested on various subtypes of nAChRs, which were heterologously expressed in *Xenopus* oocytes. nAChRs are important ligand-gated ion channels, which are widely distributed in the muscle, central nervous system and peripheral nervous system. These receptors mediate learning, feeling, nerve disorder and some severe pathologies of the brain [19]. Figure 6 (A–F) shows representative traces of rLvIA on rat α3β2, α3β4, α4β2, α6β4, α9α10 and mouse α_1_β_1_δε nAChRs. More than 90% blockade of ACh-evoked currents of rα3β2 nAChR was obtained with 1 μM rLvIA, and the blockade was rapidly reversible within one minute of toxin washout (Figure 6A). Notably, rLvIA had little or no effects for other tested nAChR subtypes at the high concentration of 10 μM (Figure 6 B–F). Concentration-response curves for rLvIA on rat and human α3β2 nAChRs are shown in Figure 7, which shows dose-dependent blockade of rat and human α3β2 receptors. The IC_50_ of rLvIA on rat α3β2 nAChR was 160.8 nM. The rLvIA also had more potent blockade for human α3β2 nAChR with an IC_50_ of 46.8 nM.

## 3. Discussion

LvIA is an α-CTx from *Conus lividus*, a marine snail native to the South China Sea of Hainan Province. α-Conotoxins are a rich source of highly selective ligands that discriminate among different nAChR subtypes [20], for example, α-CTx TxIB (α6/α3β2β3), TxID (α3β4), and Vc1.1 (α9α10) [15,17,21] *etc.* LvIA is the most selective inhibitor of α3β2 nAChR known to date, which can distinguish the α3β2 nAChR subtype from the α6β2* and α3β4 nAChR subtypes [9,22]. The α3 subunit is widely distributed in the cerebral cortex and thalamus, whereas the β2 subunit is found in the central and peripheral nervous systems. The α3β2 nAChR subtype is distributed broadly both in the central and peripheral nervous systems. Furthermore, α3β2 nAChRs in the spine have been implicated in pain transduction [23]. Therefore, obtaining enough LvIA peptide at low cost could help in the application of LvIA as a research tool to clarify the fine structure and physio-pharmacological function of the α3β2 nAChR subtype, and develop new drug leads of analgesics.

Generally, conotoxins are obtained via solid-phase chemical synthesis, which can have a high cost. This approach is also not suitable to obtain uniformly isotopically (^13^C, ^2^H, or ^15^N) labeled peptides, which are required to elucidate biophysical properties of the ligand–receptor interaction by NMR spectroscopy [24]. Another limitation of solid-phase peptide synthesis is the difficulty in synthesis and purification of some peptides, especially those of more than 30 amino acids in length. So, recombinant expression of conopeptide is a promising alternative method to chemical synthesis. In this study, a gene tandem strategy was first used to express α-CTx LvIA *in E. coli* in order to improve the stability and yield. The stability of short peptides produced in *E. coli* is improved greatly if they are fused to a carrier or linked together as large tandem repeats [25,26]. In order to enhance the stability of short conotoxins during bacterial expression, KSI was chosen as a carrier protein to produce recombinant peptides because of its highly hydrophobic character and easy formation of inclusion bodies, thus avoiding proteolytic degradation [27].

Tandem repeats recombinants of different size of α-CTx LvIA fused with KSI and a six His-tag tail were obtained successfully, which facilitated fusion protein expression and purification. The recombinant α-CTx LvIA (rLvIA) was expressed and purified effectively. The lowest yield of pure fusion protein of LvIA was 114 mg/L, which contained only one gene fragment, *i.e.*, KSI-LvIA-His_6_ fusion protein. The highest yield of recombinant fusion protein of LvIA was about 500 mg/L, which contained two or 11 gene tandem repeats, *i.e.*, KSI-LvIA_2_-His_6_ or KSI-LvIA_11_-His_6_ fusion protein. There was no obvious relationship between the number of tandem repeats and the yield of fusion protein. The fusion protein of KSI-LvIA_6_-His_6_ and KSI-LvIA_11_-His_6_ were easier to purify than the other recombinants with different number of tandem repeats. The final yields of rLvIA from KSI-LvIA_6_-His_6_ and KSI-LvIA_11_-His_6_ were ~5–6 folds higher than the others after CNBr cleavage. The yields of fusion proteins and rLvIAs were shown in Table 2.

**Table 2:** Yield of different fusion proteins of recombinant KSI(rLvIA)_*n*_His_6_ and final purified rLvIA ^a^.

KSI(rLvIA)_n_His_6_^a^	The Yield of Fusion Protein of KSI(rLvIA)_*n*_His_6_ (mg/L) ^b^	The Yield of Purified rLvIA (mg/L) ^c^
KSI(rLvIA)_1_His_6_	114.1	3.8
KSI(rLvIA)_2_His_6_	553.2	15.3
KSI(rLvIA)_3_His_6_	115.1	7.8
KSI(rLvIA)_4_His_6_	121.3	9.3
KSI(rLvIA)_5_His_6_	156.5	11.2
KSI(rLvIA)_6_His_6_	290.9	40.8
KSI(rLvIA)_11_His_6_	534.5	45.3

^a^ Fusion genes were expressed in protease deficient strain of BLR(DE3)plysS. Overnight culture was diluted 40-fold and induced with 1 mM of IPTG. Cells were harvested at 7 h after induction. The target fusion proteins were purified, dialyzed, lyophilized and weighed sequentially. ^b^ The average weights of KSI(rLvIA)_*n*_His_6_ fusion proteins were obtained from at least five separate experiments, of which the cells were harvested at 7 h after induction. ^c^ Fusion proteins of KSI(rLvIA)_*n*_His_6_ were purified by metal affinity chromatography, cleaved with CNBr, and purified by RP-HPLC according to protocols 4.4 as described in the Experimental Section. The final purified rLvIA amount of each experiment was determined by HPLC peak area with 1.5 × 10^6^ U defined as 1 nmol using Waters Empower 2 software.

The α-conotoxin LvIA was expressed in *E. coli* with tandem repeats strategy. The coding sequence of LvIA was separated by single Met residue with upstream of His_6_ tag gene and downstream of KSI gene. This strategy to express rLvIA requires that a Met residue be included in the sequence as a separator of each individual component of the fusion protein, *i.e.*, KSI-target peptides-(His-tag), in order to allow for excision of the target peptide by CNBr digestion. CNBr is a classical reagent of protein chemistry that promotes polypeptide chain hydrolysis *C*-terminally to Met residue with high specificity. The recombinant proteins were cleaved by CNBr to release fusion protein KSI, His_6_ tag and rLvIA. Another limitation of this method is that the *C*-terminal homoserine lactone residue may be generated by CNBr cleavage, which might induce unforeseen effects on bioactivity, although these adverse effects were not seen in our experiments. It is essential to use fresh reagents and to carry out the cleavage reactions under nitrogen for peptide protection.

The activity of rLvIA was investigated by electrophysiological experiments and by mouse hot-plate tests. The rLvIA was active on both rat and human α3β2 nAChRs with IC_50_ values of 160.8 nM and 46.8 nM, respectively. The rLvIA significantly increased the base pain threshold and showed analgesic effects in the mouse hot-plate test (Figure 5). The hot-plate test is a model of acute supraspinal pain, which has been employed over the last six decades to evaluate the analgesic properties of various drugs in rodents [28].

There are some limitations to producing recombinant conotoxins in *E. coli*, including a lack of post-translational modification, the addition of some extra amino acid residues at the C-terminus [29], a lack of effective cleavage reagents, and the small proportion of the conotoxin in the fusion protein, *etc.* Post-translational modifications are common in many α-CTxs, which often contain unusual amino acids, including hydroxyproline, γ-carboxyglutamic acid and bromotryptophan, as well as other modifications such as C-terminal amidation, glycosylation, *etc.* [30]. Conotoxins with these modified amino acids and other modifications cannot be synthesized in *E. coli*. All these limitations may result in a low yield of target peptides or inactive non-natural peptides [7]. However, the only post-translational modification in native LvIA is C-terminal amidation, which is not associated with modified amino acids and, thus, does not inhibit LvIA expression in *E. coli*. Previous research found that C-terminal amidation mainly played a pivotal role in the folding tendency of conopeptides [31]. The rLvIA inhibition of rat and human α3β2 nAChRs subtype was 18.5-fold or 2.7-fold less potency than native LvIA, respectively, which may be due to the Met residue addition at the N-terminus and no C-terminal amidation. Thus, rLvIA retains better activity in the human α3β2 nAChR than on the rat α3β2 nAChR. However, rLvIA had no potency in rat α3β4 nAChR (Figure 6). Native LvIA had an IC_50_ of 148 nM in rat α3β4 nAChR. Therefore, rLvIA showed better selectivity in rat α3β2 than α3β4 nAChR subtypes. In spite of some potential limitations for recombinant conotoxins, 15 conopeptides, including LvIA, had been successfully expressed in *E. coli* (Table 3). For example, Kumar GS *et al.* (2005) expressed a 13-residue acyclic conopeptide Mo1659 in *E. coli*. [32]. Furthermore, Bruce (2011) produced a 13-residue conotoxin TxVIA in yeast as well [8].

**Table 3:** Recombinant conotoxins expressed in different systems.* indicates an amidated *C*-terminus. **KSI** (ketosteroid isomerase): HTPEHITAVVQRFVAALNAGDLDGIVALFADDATVEDPVGSEPRSGTAAIREFYANSLKLPLAVELTQEVRAVANEAAFAFTVSFEYQGRKTVVAPIDHFRFNGAGKVVSIRALFGEKNIHACQM. **TRX** (thioredoxin): MSDKIIHLTDDSFDTDVLKADGAILVDFWAEWCGPCKMIAPILDEIADEYQGKLTVAKLNIDQNPGTAPKYGIRGIPTLLLFKNGEVAATKVGALSKGQLKEFLDANLA. **Cytochrome b5**: GELHPPDDRSKIAKPSETL. **Ssp DnaB intein** (*Synechocystis* sp. *dnaB* gene intein.): AISGDSLISLASTGKRVSIKDLLDEKDFEIWAINEQTMKLESAKVSRVFCTGKKLVYILKTRLGRTIKATANHRFLTIDGWKRLDELSLKEHIALPRKLESSSLQLSPEIEKLSQSDIYWDSIVSITETGVEEVFDLTVPGPHNFVANDIIVHN. SUMO (small ubiquitin-like modifier): GGSDSEVNQEAKPEVKPEVKPETHINLKVSDGSSEIFFKIKKTTPLRRLMEAFAKRQGKEMDSLRFLYDGIRIQADQTPEDLDMEDNDIIEAHREQIGG. GST (glutathione-S-transferase): MSPILGYWKIKGLVQPTRLLLEYLEEKYEEHLYERDEGDKWRNKKFELGLEFPNLPYYIDGDVKLTQSMAIIRYIADKHNMLGGCPKERAEISMLEGAVLDIRYGVSRIAYSKDFETLKVDFLSKLPEMLKMFEDRLCHKTYLNGDHVTHPDFMLYDALDVVLYMDPMCLDAFPKLVCFKKRIEAIPQIDKYLKSSKYIAWPLQGWQATFGGGDHPPKSDLEVLFQGPLGS. **FlgM** (flagellar secretion substrate): MSIDRTSPLKPVSTVQPRETTDAPVTNSRAAKTTASTSTSVTLSDAQAKLMQPGSSDINLERVEALKLAIRNGELKMDTGKIADALINEAQQDLQSN. PelB: MKYLLPTAAAGLLLLAAQPAMA. **Pro**: DDPRNGLGNLFSNAHHEMKNPEASKLNKP. **Alpha factor**: MRFPSIFTAVLFAASSALAAPVNTTTEDETAQIPAEAVIGYSDLEGDFDVAVLPFSNSTNNGLLFINTTIASIAAKEEGVSLEKREAEA.

Conotoxin	Origin	Native Peptide Sequence	Fused Partner	Recombinant Peptide	Yield (mg/L)	Activity/Target	IC50	Reference
LvIA	*C. lividus*	GCCSHPACNVDHPEIC *	KSI	rLvIA	45	Analgesic/α3β2 nAChRs	160.8 nM(rα32β)/46.8 nM(hα32β)	This work
MVIIA	*C. magus*	CKGKGAKCSRLMYDCCTGSCRSGKC	TRX	Trx-CTX MVIIA	40	analgesic function	No data	[33]
MVIIA	*C.magus*	CKGKGAKCSRLMYDCCTGSCRSGKC	GST	GST-CTX MVIIA	No data	Analgesic Activity	No data	[34]
Mol659	*C.monile*	FHGGSWYRFPWGY	Cytochrome b5	Mol659	6–8	K^+^ channel	No data	[32]
Conkunitzin-S1	*C. striatus*	KDRPSLCDLPADSGSGTKAEKRIYYNSAR KQCLRFDYTGQGGNENNFRRTYDCQRTCLYT	Ssp DnaB intein	Conk-S1	No data	K^+^ channel	1.33 nM	[35]
lt7a	*C. litteratus*	CLGWSNYCTSHSICCSGECILSYCDIW	TRX	lt7a	6	Na^+^ channel	No data	[36]
lt6c	*C. litteratus*	WPCKVAGSPCGLVSECCGTCNVLRNRCV	TRX	lt6c	12	Na^+^ channel	No data	[37]
TxVIA	*C. textile*	WCKQSGEMCNLLDQNCCDGYCIVLVCT	Alpha factor	Pro-TxVIA	10	insecticidal activity	No data	[8]
PrIIIE	*C. parius*	AARCCTYHGSCLKEKCRRKYCCG	SUMO	PrIIIE	1.5	α1β1δε/α1β1γδ	2.8 μM	[7]
Vn2	*C. ventricosus*	EDCIAVGQLCVFWNIGRPCCSGLCVFACTVKLP	GST	GST-wtCTX GST-mtCTX	No data	Toxicity to insects	No data	[38]
SIIIA	*C. striatus*	ENCCNGGCSSKWCRDHARCC	FlgM	rSIIIA	No data	Na^+^ channel	No data	[39]
GeXIVAWT	*C. generalis*	TCRSSGRYCRSPYDCRRRYCRRITDACV	PelB	rPelB-GeXIVAWT-His	61.6	cytotoxicity	No data	[40]
MrVIB	*C. marmoreus*	ACSKKWEYCIVPILGFVYCCPGLICGPFVCV	PelB	rMrVIB-His	5.9	Analgesic activity	No data	[29]
Lt15a	*C. litteratus*	ECTTKHRRCEKDEECCPNLECKCLTSPDCQSGYKCKP	TRX	Lt15a	No data	mice coma state, crouched.	No data	[41]
lt16a	*C. litteratus*	TGEDFLEECMGGCAFDFCCKR	TRX	lt16a	No data	Na^+^ channel	No data	[42]

## 4. Experimental Section

### 4.1. Materials

*E. coli* strain DH5α was from our laboratory. The BLR(DE3)pLysS strain and expression vector pET-31b(+) were from Novagen (Darmstadt, Germany). Genes encoding LvIA were synthesized by Sangon Co. (Shanghai, China). All enzymes and kits for purification of plasmid and DNA fragment were purchased from TaKaRa Co. (Dalian, China), Restriction endonuclease *Alw*NI was from NEB (Ipswich, MA, USA). Message Machine kit for cRNA synthesis was from Ambion (Austin, TX, USA). RP-HPLC analytical Vydac C18 column (5 μm, 4.6 mm × 250 mm) and preparative C18 column (10 μm, 22 mm × 250 mm) were from Grace Vydac (Hesperia, CA, USA). Ni-NTA agarose was purchased from Macherey-Nagel (Duren, Germany). Clones of rat α2-7, β2-4, as well as mouse muscle α1β1δε plasmids were kindly provided by S. Heinemann (Salk Institute, San Diego, CA, USA). Unless otherwise stated, acetylcholine chloride (ACh), isopropyl β-D-1-thiogalactopyranoside (IPTG) and other chemicals were analytical grade and purchased from Sigma (St.Louis, MO, USA).

### 4.2. Construction of Recombinant Exptession Vectors of LvIA_n_-His·Tag Genes

LvIA precursor gene was cloned from *Conus lividus* [43]. According to LvIA precursor gene sequence, a synthetic gene coding for LvIA mature peptide with sequence of GCCSHPACNVDHPEIC was designed using the preferred codon usage of *E. coli*. The oligonucleotides representing the sense and anti-sense strands of the LvIA mature peptide gene are as follows:

(1) 5’-GGTTGCTGCTCTCACCCGGCTTGCAACGTTGACCACCCGGAAATCTGCATG-3’,
(2) 3’-TACCCAACGACGAGAGTGGGCCGAACGTTGCAACTGGTGGGCCTTTAGACG-5’.

Oligos were synthesized by Sangon Co. (Shanghai, China). The two oligos (100 pmol/μL) with 1:1 ratio were denatured at 90 °C for 10 min, then annealed at 4 °C for 15 min. The annealed products at a concentration of 100 pmol/μL were ligated for 12 h with 10 units of T4 DNA ligase at 16 °C. The different tandem repeats of LvIA mature peptide gene (LvIA_n_) were analyzed by 3% agarose gel electrophoresis, and then purified, which were used to construct an array of pET-31b(LvIA)_*n*_His·Tag recombinant vectors. The entire strategy for construction and production of KSI(LvIA)_*n*_His_6_ fusion protein was shown in Figure 1. Briefly, the expression vector pET-31b(+) plasmids were prepared using a Plasmid Miniprep Kit, and were digested with *Alw*NI and dephosphorylated with CIAP. Digested vector pET-31b(+) plasmids and different length tandem repeats of LvIA_*n*_ were then ligated over 12 h with T4 DNA ligase at 16 °C. Each tandem LvIA_n_ multimer was inserted into the vector to create different recombinant expression vectors pET-31b(LvIA)_*n*_His·Tag. All constructs were sequenced to verify the number of tandem genes, which were transformed into competent cells of BLR(DE3)pLysS *E. coli* strain for protein expression.

### 4.3. Expression and Purification of KSI-LvIA_n_-His·Tag Fusion Protein

The fusion proteins of KSI-LvIA_n_-His·Tag were produced in protease deficient *E. coli* strain BLR(DE3)pLysS. A single colony of the recombinant *E. coli* was picked, then incubated overnight at 37 °C in 100 mL of Lysogeny Broth (LB) media containing 50 μg/mL ampicillin (Amp) and 34 μg/mL chloramphenicol (Chl), which was diluted by 40-fold with 1 L of fresh LB media containing Amp and Chl. The diluted *E. coli* culture was incubated in a shaker with 250 rpm at 37 °C about 2–3 h until its OD_600_ reached 0.3–0.5. The recombinant cells were induced to produced target protein by adding 1 mM IPTG. The cells were harvested by centrifuging at 10,000× *g* for 10 min at 4 °C after 7 h induction. The cell pellets were resuspended in 1× Binding Buffer (5 mM imidazole, 40 mM Tris-HCl pH 7.9, 500 mM NaCl) containing 8 M urea or 6 M guanidine hydrochloride, which were sonicated on ice until no longer viscous, and centrifuged at 12,000× *g* for 20 min at 4 °C. The levels of total and target protein were checked by SDS-PAGE. The total protein solution was recentrifuged at 12,000× *g*, 20 min, 4 °C, then the supernatant was loaded onto a 25 mL His·Bind column (Ni-NTA agarose, Macherey-Nagel, Duren, Germany). The column was washed with 50 mL same buffer containing 20 mM imidazole. The flow-through and wash fractions were collected. The fusion protein was eluted with 50 mL of 1× Elution Buffer (250 mM imidazole, 40 mM Tris-HCl pH 7.9, 500 mM NaCl, 8 M urea). The purified fusion protein was analyzed by 12% SDS-PAGE.

### 4.4. CNBr Cleavage and Purification of Recombinant Protein

The purified recombinant fusion protein solution were dialyzed over night at room temperature against 5 L ddH_2_O in 12–14 kD cutoff dialysis bags. The majority of the fusion protein formed a white precipitate and pelleted by centrifugation at 12,000× *g* for 30 min and then lyophilized as white powder. The power was weighed and dissolved in 10 mL of 70% formic acid and transferred into a flask in which 0.1 g of CNBr was added. The flask was wrapped in aluminum foil and bubbled with nitrogen for protection, which was stirred for over 12 h at room temperature to cleave the fusion protein to be recombinant α-CTx LvIA (rLvIA). The final reaction mixture was evaporated, which was resuspended in 30 mL of 40% CH_3_CN/60% H_2_O/0.1% TFA buffer at least 15 min until it was completely dissolved. The suspension of rLvIA was centrifuged at 12,000× *g* for 30 min at 4 °C. The supernatant of rLvIA was filtered with 0.22 μm filter and purified by HPLC on a reversed-phase C18 Vydac column (10 μm, 22 mm × 250 mm) using a linear gradient of acetonitrile (ACN): 0–40 min 0%–40% solvent B. Solvent B is 0.05% trifluoroacetic acid (TFA) in 90% ACN with the remainder being water. Solvent A is 0.075% TFA in H_2_O. Absorbance was monitored at 214 nm. The purity of the rLvIA was determined by analytical HPLC on a Vydac C18 column (5 μm, 4.6 mm × 250 mm) using a linear gradient of 0%–40% solvent B over 20 min. The molecular weight (MW) of the rLvIA was determined by electrospray ionization-mass spectrometer (ESI-MS, Shimadzu, Kyoto, Japan).

### 4.5. Bioassays of rLvIA in Pain Model

The rLvIA was dissolved in normal saline solution (NSS) at a concentration of 1 nmol/μL Female Kunming mice (20–25 g) were purchased from Guangdong Medical Experiment Animal Center of China with permit SCXK 2013-0002. The bioactivity of rLvIA in pain model was evaluated using IITC Hot Plate model 39 (IITC Life Science, Woodland Hills, CA, USA) at room temperature. The mice selected for pain assay, showed hind paw licking or jumping response within 5~20 s in a plexiglass cylinder with 10 cm diameter×15 cm height on the 55 ± 0.5 °C hot plate. All screened mice of 30-days-old were divided into three groups randomly. Each group consisted of eight mice (*n* = 8). One group was injected intracerebroventricular (i.c.v.) with 10 μL rLvIA solution, while another two mice groups were injected 10 μL of NSS or morphine (250 nmol) as controls by subcutaneous injection (s.c.). All the mice were individually placed on hot plate kept at a temperature of 55 ± 0.5 °C. A cut off period of 40 s was maintained to avoid paw tissue damage. The responses of hind paw licking or jumping of each mouse were recorded at 0, 30, 60, 90, 120 and 150 min after injection, respectively. The hot-plate test data were analyzed by two-way ANOVA followed by Bartlett's test for equal variances with *p* < 0.05 significance level. Statistical analysis was performed with GraphPad Prism 6.0 software (GraphPad Software Inc., La Jolla, CA, USA).

### 4.6. Electrophysiology and Data Analysis

Oocytes of *Xenopus laevis* were harvested and injected with cRNA encoding various rat, mouse or human nAChR subunits. The cRNA for each nAChR subunit was synthesized using the Message Machine kit (Ambion, Austin, TX, USA) *in vitro*, as described previously [44]. Different α and β subunits cRNA were mixed at 1:1 ratio and injected into each *Xenopus* oocyte with a Borosilicate glass needle (Sutter Instrument Co., Novato, CA, USA, O.D 1.2 mm I.D 0.69 mm). Oocytes were incubated in ND96 buffer (96.0 mM NaCl, 2.0 mM KCl, 1.8 mM CaCl_2_, 1.0 mM MgCl_2_, 5 mM HEPES, pH 7.1–7.5) at 17 °C and recorded 2–4 days post-injection. A 50 μL cylindrical oocyte chamber was gravity-perfused with ND96 containing 1 μM atropine and 0.1 mg/mL bovine serum albumin at a rate of ~2 mL/min. For α9α10 and mouse muscle α_1_β_1_δε subtype, the ND96 contained no atropine. ACh-gated currents were obtained with two-electrode voltage clamp amplifier (Axon 900A, Molecular Devices, MD, Sunnyvale, CA, USA). Currents were recorded with software Clampex 10.2 (Molecular Devices, MD, Sunnyvale, CA, USA) with holding potential of −70 mV, and sample frequency of slow 100 HZ and filter at 10 HZ. Oocytes were subjected to a 1-s pulse of 100 μM ACh in one minutes with exceptions of muscle α_1_β_1_δε and α9α10 subtypes were 10 μM. For screening the specificity of receptor, the toxin concentration was 10 μM or lower. Once a stable baseline was achieved, either ND96 alone or ND96 containing toxin was manually pre-applied for 5 min prior to the addition of the agonist. All recording were done at room temperature (24 °C). The average of three control responses just preceding a test response was used to normalize the test response to obtain % response. Each data point of the dose-response curves represents the average value ± SE of measurements from at least 4–6 oocytes. The dose-response data were fitted to the equation % response = 100/(1 + ((toxin)/IC_50_)^nH^), where nH is the Hill coefficient. All statistical results were analyzed with GraphPad Prism 6.0. IC_50_ values were determined by nonlinear regression analysis using GraphPad Prism 6.0.

## 5. Conclusions

In summary, we have provided an alternative convenient method for recombinant expression and purification of α-CTx LvIA using KSI as a carrier partner and 6× His as a purification tag. This method could prove to be a cost-effective approach not only for the production of small conopeptides but also for large conotoxins, especially for those that are difficult to synthesize by traditional chemical synthesis.

## Abbreviations

CTx	conotoxin
nAChRs	nicotinic acetylcholine receptors
KSI	ketosteroid isomerase
CNBr	cyanogen bromide
CIAP	calf intestine alkaline phosphatase
IPTG	isopropyl β- d-1-thiogalactopyranoside
RP-HPLC	reversed phase high-performance liquid chromatography
ESI-MS	electrospray ionization-mass spectrometer
SDS-PAGE	sodium dodecyl sulfate polyacrylamide gel electropheresis
TFA	trifluoroacetic acid
ACN	acetonitrile
NSS	normal saline solution
LB	Lysogeny Broth
